# Foot-and-Mouth Disease Virus Capsid Protein VP1 Interacts with Host Ribosomal Protein SA To Maintain Activation of the MAPK Signal Pathway and Promote Virus Replication

**DOI:** 10.1128/JVI.01350-19

**Published:** 2020-01-17

**Authors:** Zixiang Zhu, Weiwei Li, Xiangle Zhang, Congcong Wang, Lili Gao, Fan Yang, Weijun Cao, Kangli Li, Hong Tian, Xiangtao Liu, Keshan Zhang, Haixue Zheng

**Affiliations:** a State Key Laboratory of Veterinary Etiological Biology, National Foot and Mouth Diseases Reference Laboratory, Key Laboratory of Animal Virology of Ministry of Agriculture, Lanzhou Veterinary Research Institute, Chinese Academy of Agricultural Sciences, Lanzhou, China; Hudson Institute of Medical Research

**Keywords:** FMDV, RPSA, MAPK pathway, VP1, antagonistic mechanism

## Abstract

Foot-and-mouth disease virus (FMDV) is the causative agent of foot-and-mouth disease, a highly contagious, economically important viral disease. The structural protein VP1 plays significant roles during FMDV infection. Here, we identified that VP1 interacted with host ribosomal protein SA (RPSA). RPSA is a viral receptor for dengue virus and classical swine fever virus infections. However, the incubation of susceptible cells using the anti-RPSA antibodies did not block the infection of FMDV. Overexpression of porcine RPSA in the insusceptible cells could not trigger FMDV infection, suggesting that RPSA was not responsible for FMDV entry and infection. On the contrary, we found that overexpression of RPSA suppressed FMDV replication, and knockdown of RPSA enhanced FMDV replication. We further determined that FMDV infection activated the mitogen-activated protein kinase (MAPK) pathway and demonstrated that MAPK pathway activation was critically important for FMDV replication. RPSA negatively regulated MAPK pathway activation during FMDV infection and displayed an antiviral function. FMDV VP1 interacted with RPSA to abrogate the RPSA-mediated suppressive role in MAPK pathway activation. Together, our study indicated that MAPK pathway activation was required for FMDV replication and that host RPSA played a negatively regulatory role on MAPK pathway activation to suppress FMDV replication. FMDV VP1 bound to RPSA to promote viral replication by repressing RPSA-mediated function and maintaining the activation of MAPK signal pathway.

**IMPORTANCE** Identification of virus-cell interactions is essential for making strategies to limit virus replication and refine the models of virus replication. This study demonstrated that FMDV utilized the MAPK pathway for viral replication. The host RPSA protein inhibited FMDV replication by suppressing the activation of the MAPK pathway during FMDV infection. FMDV VP1 bound to RPSA to repress the RPSA-mediated regulatory effect on MAPK pathway activation. This study revealed an important implication of the MAPK pathway for FMDV infection and identified a novel mechanism by which FMDV VP1 has evolved to interact with RPSA and maintain the activation of the MAPK pathway, elucidating new information regarding the signal reprogramming of host cells by FMDV.

## Abstract (precis)

Identification of virus-cell interactions is essential for making strategies to limit virus replication and refine the models of virus replication. This study demonstrated that FMDV utilized the MAPK pathway for viral replication. The host RPSA protein inhibited FMDV replication by suppressing the activation of the MAPK pathway during FMDV infection. FMDV VP1 bound to RPSA to repress the RPSA-mediated regulatory effect on MAPK pathway activation. This study revealed an important implication of the MAPK pathway for FMDV infection and identified a novel mechanism by which FMDV VP1 has evolved to interact with RPSA and maintain the activation of the MAPK pathway, elucidating new information regarding the signal reprogramming of host cells by FMDV.

## INTRODUCTION

Foot-and-mouth disease virus (FMDV) is the causative agent of foot-and-mouth disease that can cause a significant epidemic disease threatening the livestock industry (1). FMDV is a member of the genus *Aphthovirus* of family *Picornaviridae*, with a single-stranded positive-sense RNA genome. The viral genome is approximately 8 kb nucleotides in length, containing a single open reading frame (∼7.7 kb) that encodes four structural proteins and eight nonstructural proteins (NSPs). The structural proteins VP1, VP2, VP3, and VP4 form the viral capsid, and the other NSPs are mainly involved in viral replication and the pathogenesis process (2). Recent studies have demonstrated that some NSPs antagonize host antiviral responses by modulating immunity (3, 4). The VP1 protein, as the main immunogenic component of FMDV particle, has also been determined to play additional roles in regulating host responses (5, 6). The interaction between VP1 and host proteins may determine the outcome of disease and pathogenesis as well. However, the host proteins that were targeted by VP1 in the viral replication cycle and the involved mechanisms are not yet fully understood.

To better understand the potential pathogenic consequences of VP1-host protein interactions, we performed a yeast two-hybrid (YTH) assay as previously described and reported that VP1 interacted with host DnaJ heat shock protein family member A3 (DNAJA3) and showed that DNAJA3 played an essential role for suppression of FMDV replication (7). In the present study, we report that FMDV VP1 interacted with host ribosomal protein SA (RPSA), a component of the ribosome (8, 9). RPSA, also known as laminin receptor 1, is ubiquitously expressed and plays multifunctional roles in various cellular responses. RPSA is involved in RNA processing (10), cell migration (11), angiogenesis (12), and human spleen development (8). It is also suggested that RPSA is involved in regulation of the mitogen-activated protein kinase (MAPK) signaling pathway correlating with tumor dissemination (13). However, whether it includes a regulatory function during viral replication remains unknown. The MAPK signaling cascade regulates a wide variety of physiological processes, such as cell differentiation, survival, growth, cancer-immune evasion, and apoptotic cell death (14, 15). Both extracellular and intracellular stimuli, such as cytokines, hormones, oxidative stress, and virus infection, can activate MAPK pathways (16 – 18).

Deviation from the well-balanced control of MAPK signaling cascade has been implicated in many virus infections. For example, Ebola virus persistently infects and escapes from cells through activation of the Ras/MAPK pathway (19). Influenza A virus-induced MAPK signaling cascade results in a large amount of cytokines production and airway inflammation (20). Infectious salmon anemia virus activates MAPK pathway to favor the synthesis of new viral progeny by promoting cellular apoptosis (21). RPSA is involved in regulation of MAPK signaling pathway. Here, VP1 interacted with RPSA, which implied a potential role of FMDV in manipulating the MAPK signal pathway.

The VP1-RPSA interaction initially identified by the YTH assay was confirmed by a coimmunoprecipitation assay. We further showed that the overexpression of RPSA decreased FMDV replication, and knockdown of RPSA enhanced FMDV replication within FMDV-infected cells. FMDV infection induced the activation of MAPK signaling cascade. Suppression of the MAPK signal pathway by the MAPK pathway-specific inhibitor U1026 significantly impaired FMDV replication. RPSA played an essential role in suppression of the MAPK signal pathway activation during FMDV infection. Overexpression of FMDV VP1 impaired the RPSA-mediated suppressive effect on the MAPK signal pathway activation. Overexpression of VP1 truncated mutant with abolished ability to bind with RPSA showed no effect on MAPK signal pathway activation, supporting the possibility that the interaction between VP1 and RPSA contributed to the increased MAPK signaling and enhanced viral replication within FMDV-infected cells.

## RESULTS

### FMDV structural VP1 protein interacted with porcine host protein RPSA.

**FIG 1:**
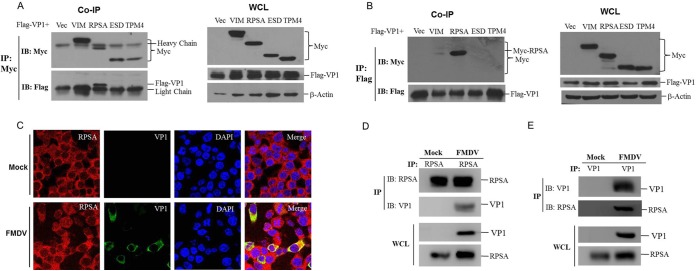
FMDV VP1 interacted with host RPSA protein. (A and B) HEK-293T cells were cotransfected with 5 μg of Myc-vector-, Myc-VIM-, Myc-RPSA-, Myc-ESD-, or Myc-TPM4-expressing plasmids, and 5 μg of Flag-VP1-expressing plasmids for 36 h. The cells were then lysed and immunoprecipitated by anti-Myc antibody (A) or anti-Flag antibody (B). The immunoprecipitation (IP) complexes and whole-cell lysates (WCLs) were subjected to Western blotting using an anti-Myc, anti-Flag, or anti-β-actin antibody. (C) PK-15 cells were mock infected or infected with FMDV for 12 h, the subcellular localization of RPSA and FMDV VP1 was analyzed by immunofluorescence assay. Anti-VP1 (green) and anti-RPSA (red) antibodies and DAPI (blue) were used to stain the cells. (D and E) PK-15 cells were mock infected or infected with FMDV for 12 h. The cell lysates were then immunoprecipitated by anti-RPSA (D) or anti-VP1 (E) antibody. The IP complexes and WCLs were subjected to Western blotting with the anti-RPSA and anti-VP1 antibodies.

Cellular RPSA was identified as a potential target of FMDV VP1 by YTH assay in our previous study (7). To confirm the VP1-RPSA interaction, we performed the coimmunoprecipitation assay. Another VP1-targeted candidate host protein, vimentin (VIM), screened by YTH assay and two VP1-nonbinding proteins, esterase D (ESD) and tropomyosin 4 (TPM4), were also analyzed in the experiment. Human embryonic 293T (HEK-293T) cells were cotransfected with Myc-vector-, Myc-VIM-, Myc-RPSA-, Myc-ESD-, or Myc-TPM4-, and Flag-VP1-expressing plasmids. The cell lysates were immunoprecipitated with a mouse monoclonal anti-Myc antibody, and the coprecipitated proteins were analyzed by immunoblotting with a mouse monoclonal anti-Flag antibody. We found that RPSA efficiently pulled down VP1; however, VIM, ESD, and TPM4 did not pull down VP1 (Fig. 1A). A reverse immunoprecipitation using a rabbit polyclonal anti-Flag antibody was also performed. Similarly, VP1 pulled down RPSA, and VP1 did not pull down VIM, ESD, and TPM4 (Fig. 1B). These results suggested that VP1 interacted with RPSA. To confirm that the VP1-RPSA interaction occurs in FMDV-infected cells, the subcellular localization of VP1 and RPSA in FMDV-infected cells was evaluated. The colocalization of VP1 and RPSA was clearly observed in the cytoplasm of FMDV-infected PK-15 cells (Fig. 1C). The coimmunoprecipitation assay was also performed using the lysates from FMDV-infected cells. RPSA clearly pulled down FMDV VP1 protein in the context of viral infection (Fig. 1D). The reverse immunoprecipitation assay was similarly performed using the anti-VP1 antibody, and the results showed that VP1 also immunoprecipitated RPSA (Fig. 1E). Together, these results suggested that FMDV VP1 interacted with host RPSA protein.

### RPSA was not a host receptor for FMDV infection.

**FIG 2:**
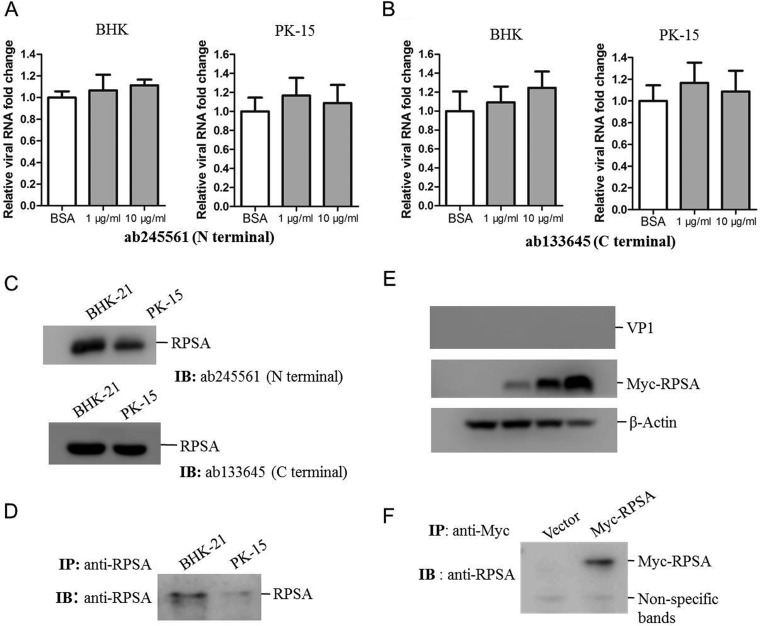
RPSA was not responsible for FMDV entry. (A and B) BHK-21 and PK-15 cells were incubated with 10 μg/ml of BSA or 1 or 10 μg/ml of the anti-N terminus (A) or anti-C terminus (B) of RPSA antibodies for 1 h and then infected with FMDV (MOI of 0.5) for 12 h. The FMDV RNA levels were measured by qPCR. (C) BHK-21 and PK-15 cells were collected and lysed, respectively. The expression of RPSA was detected by using the two anti-RPSA antibodies. (D) The cell membrane proteins were extracted from the BHK-21 and PK-15 cells. The membrane fractions were then immunoprecipitated with anti-RPSA antibody and subjected to Western blotting. (E) HEK-293T cells were transfected with 0, 0.5, 1, or 2 μg of Myc-RPSA-expressing plasmids for 24 h. The cells were then infected with FMDV at an MOI of 1 for 12 h. The cell lysates were subjected to Western blotting with anti-VP1, anti-Myc, and anti-β-actin antibodies. (F) HEK-293T cells were transfected with 2 μg of vector or Myc-RPSA-expressing plasmids for 36 h. The cell membrane proteins were immunoprecipitated with anti-Myc antibody and subjected to Western blotting.

RSPA is ubiquitously distributed in host cells, and it is also expressed on the cell surface. RPSA is a viral receptor for dengue virus and classical swine fever virus (22, 23). Here, we showed that FMDV VP1 interacted with host RPSA in the context of viral infection. To investigate whether RPSA is a potential receptor for FMDV, we performed an antibody inhibition assay. RPSA spans the plasma membrane once with its C terminus exposed to the extracellular space (24). We used two antibodies against the N terminus or C terminus of RPSA for the inhibition assay. Baby hamster kidney (BHK-21) or porcine kidney (PK-15) cells were preincubated with the antibody against the N terminus or C terminus of RPSA, and the cells were then infected with FMDV. The results indicated that neither antibody had an inhibitory effect on FMDV infection at a concentration of 1 or 10 μg/ml (Fig. 2A and B). Whether FMDV used some other alternative receptors during the blocking process remained unknown. The present data suggested that blocking of RPSA had no significant interference on FMDV entry. The expression of RPSA in BHK-21 and PK-15 cells was confirmed by immunoblotting with both the N-terminus and C-terminus RPSA antibodies, which showed that RPSA could be detected from both BHK-21 and PK-15 cells using the two antibodies (Fig. 2C). The cellular membrane fractions of BHK-21 and PK-15 cells were extracted, and the expression of RPSA in the membrane fractions was also confirmed (Fig. 2D). We further overexpressed porcine RPSA in HEK-293T cells, which are not susceptible to FMDV infection. HEK-293T cells were transfected with increasing amounts of Myc-RPSA plasmids for 24 h and then infected with FMDV for 12 h. The cells were subjected to Western blotting to evaluate the viral infection status in the cells. The overexpression of RPSA did not cause the virus replication in HEK-293T cells (Fig. 2E). We also determined that overexpression of RPSA did not promote the virus attachment to HEK-293T cells (data not shown). The expression of Myc-RPSA in the cellular membrane fractions of HEK-293T cells was also confirmed by Western blot analysis (Fig. 2F). These results indicated that RPSA was not a receptor for FMDV infection.

### RPSA inhibited FMDV replication during virus infection.

**FIG 3:**
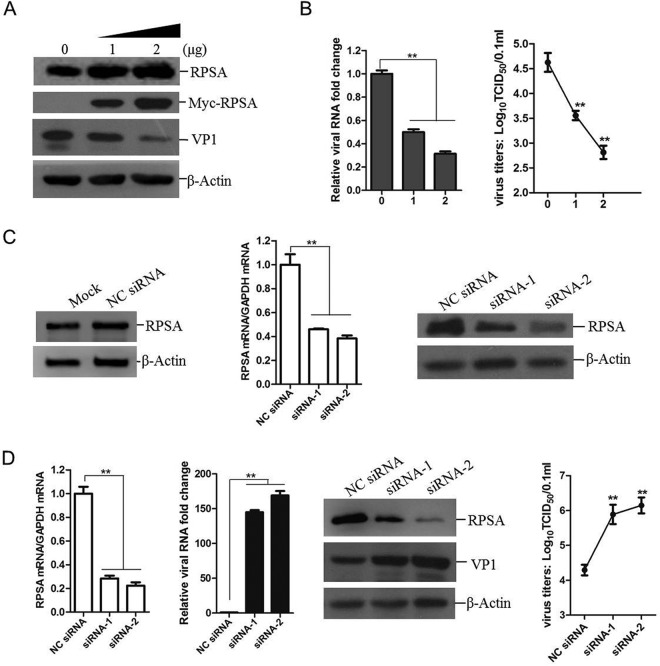
RPSA performed an antiviral role during FMDV replication. (A and B) PK-15 cells were transfected with 0, 1, or 2 μg of Myc-RPSA-expressing plasmids for 24 h, followed by infection with FMDV at an MOI of 0.5 for 12 h. (A) The cells were subjected to Western blotting with anti-RPSA, anti-Myc, anti-VP1, and anti-β-actin antibodies. (B) FMDV RNA levels were detected using qPCR, and the viral yields were determined by using a 50% tissue culture infective dose (TCID_50_) assay. (C) PK-15 cells were mock transfected or transfected with 120 nM NC siRNA or RPSA siRNA (siRNA-1 or siRNA-2) for 36 h. The knockdown efficiency was then determined by qPCR and Western blotting. (D) PK-15 cells were transfected with NC siRNA, siRNA-1, or siRNA-2 for 36 h, followed by infection with FMDV at an MOI of 0.5 for 12 h. The mRNA expression levels of RPSA and FMDV were then measured by qPCR. The protein expression levels of RPSA and FMDV VP1 proteins were detected by Western blotting. The FMDV yields were determined by a TCID_50_ assay.

To access the role of RPSA during FMDV infection, we evaluated FMDV replication level in cells overexpressing RPSA. Porcine kidney (PK-15) cells were transfected with different amounts of RPSA-expressing plasmids for 24 h; empty vector plasmids were used in the transfection process to ensure the cells received same amounts of total plasmids. The transfected cells were then infected with FMDV for another 12 h. The expression of viral proteins was then detected by Western blotting. A considerable reduction of viral proteins expression was observed in the RPSA-overexpressing cells compared to cells transfected with vector plasmids. The total expression of RPSA in the virus-infected cells was measured, and this showed that RPSA inhibited the viral replication in a dose-dependent manner (Fig. 3A). The viral RNA in the cells and the viruses in the cell culture supernatant were also measured. As expected, both the viral RNA and virus yields significantly decreased in the RPSA-overexpressing cells compared to vector-transfected cells (Fig. 3B). Furthermore, the viral RNA, viral protein, and virus yields in the RPSA knockdown cells were measured. The knockdown of RPSA was performed by transfection of small interference RNA (siRNA) that targets RPSA. PK-15 cells were transfected with the negative-control siRNA (NC siRNA) or RPSA siRNA for 36 h, and the expression of endogenous RPSA was assessed by Western blotting to validate the silencing efficiency of the siRNAs used in the present study. Transfection of NC siRNA did not affect the basal expression of RPSA protein. The synthesized siRNA-1 and siRNA-2 efficiently silenced both mRNA and protein expression of RPSA (Fig. 3C). PK-15 cells were transfected with siRNA-1 or siRNA-2 for 36 h and then infected with equal amounts of FMDV for 12 h. The expression of RPSA was detected, and the viral RNA, viral protein, and virus yields were measured, respectively. Knockdown of RPSA caused remarkable increase in viral RNA, viral protein expression, and virus yields (Fig. 3D). These results indicated that RPSA played an antiviral role during FMDV infection.

### FMDV infection activated MAPK signal pathway to promote virus replication.

**FIG 4:**
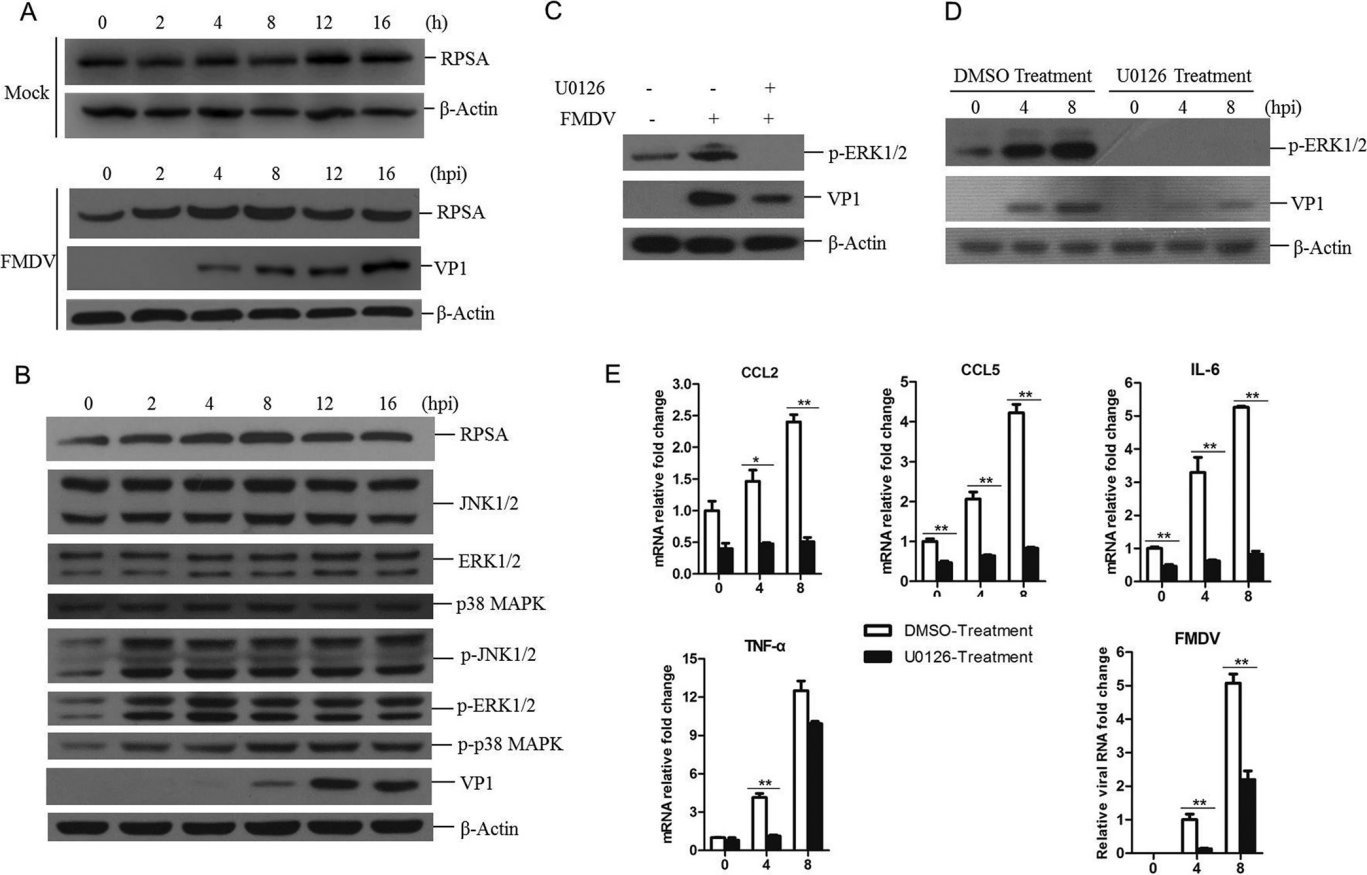
The activation of MAPK signaling pathway was essential for FMDV replication. (A and B) PK-15 cells were mock infected or infected with FMDV at an MOI of 0.5 for 0, 2, 4, 8, 12, or 16 h. The cells were then lysed and subjected to Western blotting. (A) The expression of RPSA and VP1 proteins was detected by using anti-RPSA and anti-VP1 antibodies, respectively. (B) The expression levels of MAPK pathway related proteins, including JNK1/2, ERK1/2, and p38 kinases and phosphorylated JNK1/2, ERK1/2, and p38 kinases (p-JNK1/2, p-ERK1/2, and p-p38), were detected by Western blotting. (C) PK-15 cells were pretreated with DMSO (solvent control) or 20 μM U0126 for 1 h and then infected with FMDV for 8 h. The expression levels of p-ERK1/2 and FMDV VP1 proteins were detected by Western blotting. (D and E) PK-15 cells were pretreated with DMSO or U0126 for 1 h and then infected with FMDV for 0, 4, or 8 h. (D) The expression levels of p-ERK1/2 and FMDV VP1 proteins were detected by Western blotting. (E) The mRNA expression levels of CCL2, CCL5, IL-6, TNF-α, and FMDV were measured by qPCR.

As mentioned previously, RPSA is a multifunctional protein. How RPSA inhibits FMDV replication remains unknown. To uncover the involved mechanism of RPSA-mediated anti-FMDV effect, we first explored the expression of RPSA in FMDV-infected cells. PK-15 cells were infected with FMDV and collected at 0, 2, 4, 8, 12, or 16 hpi, and then the cells were subjected to Western blotting. The basal expression level of RPSA in the mock-infected cells was also evaluated. No remarkable change of RPSA protein abundance was observed in the mock-infected cells at different times. A gradual increase in RPSA protein abundance was observed as infection progressed at the early infection period (from 2 to 8 h postinfection [hpi]), and the abundance remained almost unchanged after 8 hpi (from 8 to 16 hpi) (Fig. 4A). This indicated that the expression of RPSA was upregulated at the early stage during FMDV infection. The status of MAPK signal pathway during FMDV infection was also investigated. The degrees of total and phosphorylated ERK, JNK, and p38 kinases at 0, 2, 4, 8, 12, and 16 hpi were examined by Western blotting. We found that MAPK signaling was activated in FMDV-infected cells. As shown in Fig. 4B, FMDV infection remarkably triggered the phosphorylation of JNK1/2, ERK1/2, and p38, and the phosphorylation of JNK1/2, ERK1/2, and p38 started at 2 hpi, indicating that all of these three MAPKs were activated in response to FMDV infection.

It has been reported that MAPKs are phosphorylated during various virus infection and that the activation of MAPK pathway promotes the replication of many viruses (17, 25, 26). The role of MAPK pathway during FMDV infection remains unknown. To explore the effect of MAPK activation on FMDV replication, a chemically synthesized organic compound U0126 that specifically inhibits the activation of MAPK (ERK1/2) was used, as previously described (27). An MTT assay was performed to evaluate the compound on cell viability which showed that there was no significant effect on the viability of PK-15 cells treated with 20 μM U0126 (data not shown). PK-15 cells were pretreated with U0126 and then infected with FMDV. Pretreatment of U0126 significantly suppressed ERK1/2 phosphorylation and resulted in dramatically decreased replication of FMDV (Fig. 4C). A time course analysis further confirmed that FMDV infection activated MAPK signaling and pretreatment of U0126 considerably blocked FMDV replication (Fig. 4D). MAPK activation plays a critical role in mediating proinflammatory cytokine production (28). To evaluate the expression of proinflammatory cytokines in FMDV-infected cells pretreated with U0126, the total RNA was extracted from infected cells pretreated with dimethyl sulfoxide (DMSO) control or U0126. The transcripts of CCL2, CCL5, interleukin-6 (IL-6), tumor necrosis factor alpha (TNF-α), and FMDV were measured by qPCR. We found that the expression of CCL2, CCL5, IL-6, and TNF-α was inhibited through the course of infection in U0126-pretreated cells and that viral RNA abundance was also decreased in U0126-pretreated cells (Fig. 4E). This suggested that MAPK signaling played a key role on FMDV replication in PK-15 cells and that the upregulation of proinflammatory cytokines was involved in this process.

### RPSA inhibits MAPK signaling in FMDV-infected cells to suppress viral replication.

**FIG 5:**
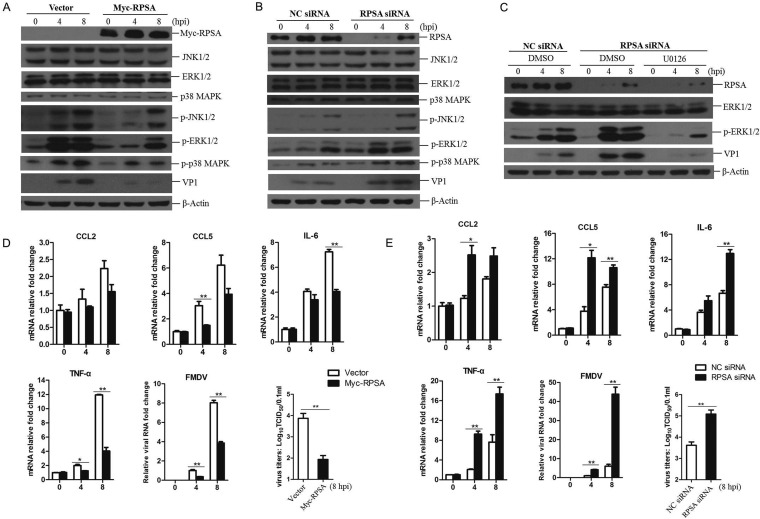
RPSA inhibited the activation of MAPK pathway during FMDV infection. (A) PK-15 cells were transfected with 2 μg of Myc-RPSA-expressing plasmids or empty vector for 24 h and then infected with FMDV for 0, 4, or 8 h. The expression levels of Myc-RPSA, JNK1/2, ERK1/2, and p38 kinases p-JNK1/2, p-ERK1/2, p-p38, and FMDV VP1 proteins were detected by Western blotting. (B) PK-15 cells were transfected with NC siRNA or RPSA siRNA (siRNA-2) for 36 h, followed by infection with FMDV for 0, 4, or 8 h. The expression levels of RPSA, JNK1/2, ERK1/2, and p38 kinases and p-JNK1/2, p-ERK1/2, p-p38, and FMDV VP1 proteins were detected by Western blotting. (C) PK-15 cells were transfected with NC siRNA or RPSA siRNA for 36 h and then treated with DMSO or U0126 for 1 h. The cells were infected by FMDV for 0, 4, or 8 h. The expression levels of RPSA, ERK1/2, p-ERK1/2, and FMDV VP1 proteins were detected by Western blotting. (D) PK-15 cells were transfected with 2 μg of Myc-RPSA-expressing plasmids or empty vector for 24 h and then infected with FMDV for 0, 4, or 8 h. The mRNA expression levels of CCL2, CCL5, IL-6, TNF-α, and FMDV were measured by qPCR. The FMDV yields at 8 hpi were determined by a TCID_50_ assay. (E) PK-15 cells were transfected with NC siRNA or RPSA siRNA for 36 h, followed by infection with FMDV for 0, 4, or 8 h. The mRNA expression levels of CCL2, CCL5, IL-6, TNF-α, and FMDV were measured by qPCR. The FMDV yields at 8 hpi were determined by a TCID_50_ assay.

RPSA is involved in regulation of MAPK signaling pathway correlating with tumor dissemination (13). However, little is known about the association of RPSA with MAPK signaling in virus-infected cells. To investigate whether RPSA regulates MAPK signaling during FMDV infection, PK-15 cells were transfected with RPSA-expressing plasmids or empty vector for 24 h and then infected with FMDV. The expression of total and phosphorylated MAPKs was examined by Western blotting. The upregulation of RPSA significantly suppressed the phosphorylation of JNK1/2, ERK1/2, and p38 induced by FMDV, and the viral replication was also decreased (Fig. 5A). This suggested that RPSA suppressed the activation of MAPKs signal pathway to inhibit FMDV replication. The regulative role of RPSA on MAPK signaling in FMDV-infected cells was also investigated in the RPSA knockdown cells. PK-15 cells were transfected with RPSA siRNA to silence the expression of RPSA, and the NC siRNA was used as a control. The transfected cells were infected with FMDV at 36 h posttransfection for 0, 4, or 8 h, and the state of MAPKs signaling was evaluated. Knockdown of RPSA remarkably promoted the phosphorylation of ERK1/2, JNK1/2, and p38 during FMDV infection. As expected, FMDV replication was considerably enhanced in the RPSA knockdown cells (Fig. 5B).

Knockdown of RPSA resulted in highly enhanced MAPK phosphorylation and viral replication in FMDV-infected cells. We further investigated whether this enhancive effect could be blocked by incubation of U0126. The results showed that incubation of U0126 clearly blocked MAPKs phosphorylation in RPSA knockdown cells during FMDV infection, and the viral replication was also strikingly decreased (Fig. 5C). The silence of RPSA enhanced MAPK signaling and FMDV replication, and this enhancive effect can be blocked by treatment of U0126. This indicated that the activation of MAPKs signal pathway was essential for FMDV replication, and RPSA performed a suppressive role on the activation of the MAPK signal pathway to inhibit FMDV replication.

The activation of the MAPK signal pathway by virus infection results in the expression of a large amount of proinflammatory cytokines. To confirm the regulative role of RPSA on the MAPK signal pathway activation during FMDV infection, the expression of proinflammatory cytokines in FMDV-infected RPSA-overexpressing cells and RPSA knockdown cells was subsequently evaluated. The results showed that the expression of CCL5, IL-6, and TNF-α was impaired by the upregulation of RPSA, accompanied by a decrease in FMDV replication and yields (Fig. 5D). In contrast, the expression of CCL2, CCL5, IL-6, and TNF-α were considerably enhanced in RPSA knockdown cells, with an increased FMDV replication and yields (Fig. 5E). Together, these results indicated that RPSA inhibited MAPK signal pathway activation in FMDV-infected cells to suppress viral replication.

### FMDV VP1 interacted with RPSA to repress the RPSA-mediated effect on the regulation of MAPK signaling.

**FIG 6:**
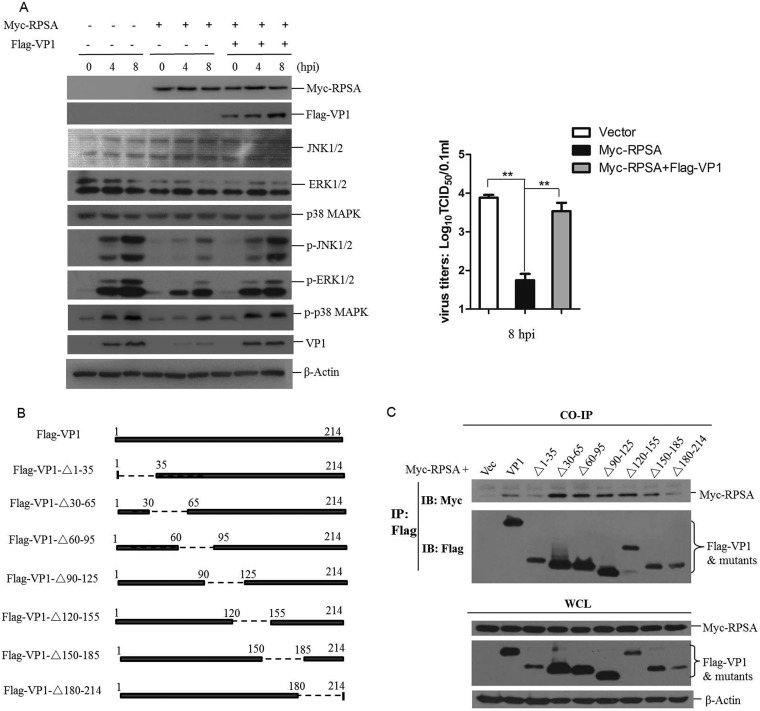

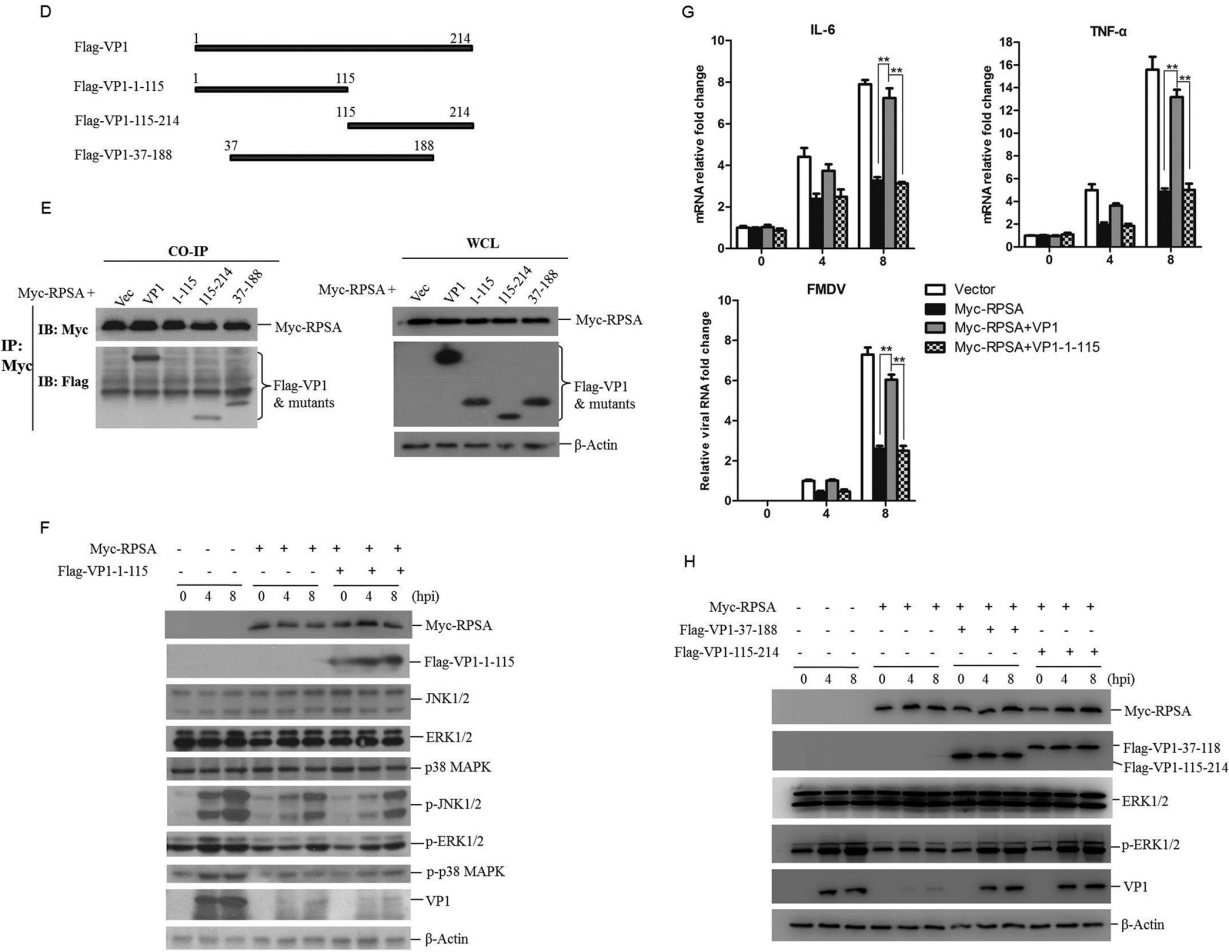
FMDV VP1 interacted with RPSA to antagonize RPSA-mediated suppressive effect on MAPK signal pathway activation. (A) PK-15 cells were cotransfected with Myc vector or Myc-RPSA-expressing plasmids and Flag vector- or Flag-VP1-expressing plasmids, followed by infection with FMDV for 0, 4, or 8 h. The expression levels of Myc-RPSA, Flag-VP1, JNK1/2, ERK1/2, and p38 kinases and p-JNK1/2, p-ERK1/2, p-p38, and FMDV VP1 proteins were detected by Western blotting. The FMDV yields at 8 hpi were determined by a TCID_50_ assay. (B) Schematic presentation of a series of VP1 truncated mutants. (C) HEK-293T cells were cotransfected with 5 μg of Myc-RPSA and 5 μg of Flag vector, Flag-VP1, or Flag-tagged VP1 truncated mutants expressing plasmids for 36 h. The cells were then lysed and immunoprecipitated by anti-Flag antibody. The IP complexes and WCLs were subjected to Western blotting. (D) Schematic presentation of a series of VP1 truncated mutants. (E) HEK-293T cells were cotransfected with 5 μg of Myc-RPSA and 5 μg of Flag vector, Flag-VP1, or Flag-tagged VP1 truncated mutants expressing plasmids for 36 h. The cells were then lysed and immunoprecipitated with anti-Myc antibody. The IP complexes and WCL were subjected to Western blotting. (F) PK-15 cells were cotransfected with Myc vector- or Myc-RPSA-expressing plasmids and Flag vector- or Flag-VP1-1-115-expressing plasmids, followed by infection with FMDV for 0, 4, or 8 h. The expression levels of Myc-RPSA, Flag-VP1-1-115, JNK1/2, ERK1/2, and p38 kinases and p-JNK1/2, p-ERK1/2, p-p38, and FMDV VP1 proteins were detected by Western blotting. (G) PK-15 cells were cotransfected with Myc vector- or Myc-RPSA-expressing plasmids and Flag vector-, Flag-VP1-, or Flag-VP1-1-115-expressing plasmids, followed by infection with FMDV for 0, 4, or 8 h. The mRNA expression levels of IL-6, TNF-α, and FMDV were measured by qPCR. (H) PK-15 cells were cotransfected with Myc vector- or Myc-RPSA-expressing plasmids and Flag vector-, Flag-VP1-37-188-, or Flag-VP1-115-214-expressing plasmids, followed by infection with FMDV for 0, 4, or 8 h. The expression levels of Myc-RPSA, Flag-VP1-37-188, Flag-VP1-115-214, ERK1/2, p-ERK1/2, and FMDV VP1 proteins were detected by Western blotting.

As described above, FMDV VP1 interacted with RPSA. To evaluate the role of the VP1-RPSA interaction on MAPK signaling during FMDV replication, the expression of total and phosphorylated MAPKs in RPSA-overexpressing cells was evaluated in the absence or presence of Flag-VP1 protein. We found that the overexpression of VP1 restored the phosphorylation of ERK1/2, JNK1/2, and p38 which had been suppressed by RPSA. As shown in Fig. 6A, the overexpression of RPSA significantly suppressed ERK1/2, JNK1/2, and p38 phosphorylation at 4 and 8 hpi. However, this inhibitive effect was considerably impaired by the overexpression of VP1. The restored phosphorylation of MAPKs in turn resulted in increased FMDV replication (Fig. 6A).

To identify the region of FMDV VP1 that was responsible for the VP1-RPSA interaction, a series of Flag-tagged truncated VP1 constructs were constructed (Fig. 6B). Myc-RPSA and Flag-VP1 or Flag-VP1 truncated mutants were cotransfected into HEK-293T cells. The interaction status was investigated by a coimmunoprecipitation assay. The results showed that all of the truncated mutants could bind to RPSA, suggesting that multiple regions might be involved in VP1-RPSA interaction (Fig. 6C). We further constructed three Flag-tagged truncated VP1 constructs that included longer regions of VP1 (Fig. 6D). The interaction between the VP1 mutants and RPSA was further investigated. The N-terminal region of VP1 from amino acids 1 to 115 did not bind to RPSA, and the regions of VP1 from amino acids 37 to 188 and amino acids 115 to 214 bound to RPSA (Fig. 6E). The truncated mutant 1–180 (VP1-Δ180-214) could bind to RPSA (Fig. 6C). These results suggested that there was no binding site in the 1–115 region of VP1 and that there was at least one binding site or region located in the 115–180 region of VP1.

The VP1-1-115 mutant did not interact with RPSA. To investigate whether this region also lost the ability to regulate the MAPK signal pathway during FMDV infection, the effect of VP1-1-115 on MAPK signaling in FMDV-infected RPSA-overexpressing cells was evaluated. The results showed that overexpressing Flag-VP1-1-115 did not restore the phosphorylation of ERK1/2, JNK1/2, and p38 that had been suppressed by RPSA (Fig. 6F). Meanwhile, the transcripts of IL-6, TNF-α, and FMDV were investigated in FMDV-infected RPSA-overexpressing cells in the presence or absence of Flag-VP1 or Flag-VP1-1-115. The wild-type VP1 protein enhanced IL-6 and TNF-α expression, leading to an increased replication of FMDV. However, the VP1-1-115 mutant did not enhance the expression IL-6 and TNF-α and also failed to promote FMDV replication (Fig. 6G). The effect of VP1-37-188 and VP1-115-214 mutants on MAPK signaling in FMDV-infected RPSA-overexpressing cells was further evaluated. The results showed that overexpression of either VP1-37-188 or VP1-115-214 mutants restored the phosphorylation of ERK1/2 and abolished Myc-RPSA-mediated antiviral activity against FMDV (Fig. 6H). These results suggested that VP1 interacted with RPSA to restore the activation of MAPKs signaling and promote FMDV replication.

## DISCUSSION

FMDV utilizes various strategies to counteract host antiviral responses for its replication (4). However, the involved mechanisms are not fully understood. One potential strategy that FMDV may use to counteract host antiviral response involves interaction with host proteins, enabling the virus to impair the function of host protein and subverting natural signal transduction in favor of viral replication. In our previous work, we determined that FMDV 2B protein interacted with cellular RIG-I and LGP2 to suppress host antiviral responses, promoting viral replication (3, 29). Here, we identified that the FMDV structural protein VP1 interacted with the host RPSA protein, a multifunctional and ubiquitously expressed host protein that regulates various physiological processes.

VP1 is an important structural protein of picornavirus that reveals the ability to bind to various host proteins. For example, VP1 of enterovirus 71 (EV71) interacts with host annexin II on the cell surface to enhance viral entry and infectivity (30). FMDV VP1 interacts with host soluble resistance-related calcium-binding protein to inhibit the type I interferon response in favor of viral replication (5). FMDV VP1 is extensively involved in the regulation of FMDV infection and replication. The amino acid sequence RGD at positions 145 to 147 and the C-terminal region of VP1-203-213 are involved in cell attachment by binding to host integrins (31, 32). Moreover, VP1 inhibits IRF3 phosphorylation to impair type I interferon production and promote virus replication. The host DNAJA3 protein also interacts with the C-terminal 188–211 region of VP1 to induce the lysosomal degradation of VP1 and attenuate VP1-mediated suppressive effect on interferon production (7). The 135–144 region of FMDV VP1 contains both B- and T-cell epitopes and has been used as an immunogen in hosts (33). RPSA is identified as a receptor for several viruses, including adeno-associated virus serotypes 2, 3, 8, and 9 and dengue virus (23, 34). However, in the present study, we found that incubation of the cells using the anti-RPSA antibodies did not block the infection of FMDV. The 115–180 region of VP1 was involved in RPSA-VP1 interaction. Although this region also includes the RGD sequence that is responsible for cell attachment by FMDV, deletion of the 120–155 region of VP1 that contains the RGD sequence did not affect RPSA-VP1 interaction. We suggest that RPSA is not a cellular receptor for FMDV entry. In contrast, overexpression of RPSA resulted in a decreased replication of FMDV, and knockdown of RPSA increased FMDV replication. RPSA-VP1 interaction did not affect the viral entry, and it played an antiviral function during FMDV infection. The 115–180 region of VP1 did not overlap the binding region for DNAJA3, indicating that RPSA was not involved in DNAJA3-induced degradation of VP1.

RPSA induces the dephosphorylation of kinases ERK, JNK, and p38 and decreases the activity of the three MAPK cascades in tumor cell lines (13). The MAPK pathway is activated and manipulated by diverse group of viruses during viral infection (35). However, little is known about the MAPK signal pathway in FMDV-infected cells. Excessive inflammation is becoming accepted as a critical factor in many viral infection diseases. MAPKs are important regulators of inflammatory cytokine and chemokine expression, and many viruses have been shown to exploit MAPK signaling by causing excessive inflammation for their own replication (20, 28, 35). Excessive inflammatory response has been associated with FMDV-induced diseases (36 – 38). A recent study reported that in evolved BHK-21 cell lines that were insensitive to FMDV infection, the MAPK signaling was weaker than in wild-type BHK-21 cells, suggesting that the decreased MAPK signaling may contribute to the resistance of cells to FMDV infection (39). In the present study, we demonstrated that FMDV infection activated the MAPK signal pathway, and we further showed that the MAPK signal pathway positively regulated FMDV replication. Treatment of cells with the specific inhibitors of MAPK signal pathway U0126 significantly blocked MAPK signaling and suppressed FMDV replication. This confirmed that the MAPK pathway is manipulated by FMDV and that the MAPK signal pathway might be a potential target for the suppression of FMDV infection.

RPSA repressed MAPK signaling in PK-15 cells; therefore, it is easily understood that the overexpression of RPSA suppressed FMDV replication and the knockdown of RPSA enhanced FMDV replication. RPSA performed its antiviral function against FMDV by the suppression of MAPK signal pathway activation. The MAPK signal pathway is also required for EV71 replication (40). Whether RPSA also suppresses EV71 and other picornaviruses remains unknown. Moreover, the mechanism by which RPSA suppresses MAPK signaling should further be investigated.

It is widely known that FMDV becomes an effective pathogen by evading the host immune response. Various FMDV proteins have been shown to interact with host proteins to suppress host antiviral response, including VP1 protein (4). Here, we identified that VP1 interacted with RPSA to impair the inhibitive effect of RPSA on MAPK signal pathway activation. VP1-RPSA interaction enhanced MAPK signaling and promoted FMDV replication. FMDV infection causes excessive inflammation in the oral cavity and the coronary bands (38); the unbalanced activation of MAPK signaling might be involved in the excessive inflammation, and VP1 protein might be one of the viral factors responsible for induction of the disease.

In conclusion, the present study determined the positive regulatory role of the MAPK signal pathway in FMDV replication and demonstrated the antiviral role of RPSA by repressing MAPK signaling during FMDV infection. It also showed a novel mechanism by which FMDV VP1 has evolved to interact with RPSA and counteract the RPSA-mediated antiviral effect.

## MATERIALS AND METHODS

### Cell lines and reagents.

PK-15, BHK-21, and HEK-293T cells were maintained in Dulbecco modified Eagle medium (Invitrogen, Carlsbad, CA) supplemented with 10% heat-inactivated fetal bovine serum (FBS) and 1% penicillin-streptomycin and then cultured at 37°C under 5% CO_2_. PK-15 and BHK-21 cells are susceptible to FMDV infection and were used for viral infection experiments in the present study. HEK-293T cells are easily transfected and have been extensively used as an expression tool for host or viral proteins and used to investigate protein-protein interactions. We also used HEK-293T cells to perform the coimmunoprecipitation assays and identify the protein-protein interactions. The commercial antibodies used in this study were as follows: anti-Flag M2 antibody produced in mice (Sigma, St. Louis, MO), anti-Flag antibody produced in rabbits (Sigma), anti-β-actin mouse antibody (Thermo Scientific, Waltham, MA), MAPK family antibody sampler kit (Cell Signaling Technology, Beverly, MA), anti-phospho-p44/42 MAPK (Erk1/2) (Thr202/Tyr204) rabbit antibody (Cell Signaling Technology), anti-phospho-p38 MAPK (Thr180/Tyr182) rabbit antibody (Cell Signaling Technology), anti-phospho-SAPK/JNK (Thr183/Tyr185) rabbit antibody (Cell Signaling Technology), anti-RPSA rabbit antibodies (ab245561 and ab133645; Abcam, Cambridge, MA), anti-RPSA mouse antibody (MyBioSource, San Diego, CA), and anti-Myc mouse antibody (Santa Cruz Biotechnology, Santa Cruz, CA). Anti-VP1 antibody was provided by OIE FMD reference laboratory of China (Lanzhou, Gansu, People’s Republic of China). The MAPK signal pathway inhibitor U0126 was purchased from Cell Signaling Technology.

### Viruses and viral infection.

The FMDV type O strain O/BY/CHA/2010 (GenBank accession number JN998085) was used for viral challenge experiments in this study. O/BY/CHA/2010 was obtained from the National Foot and Mouth Diseases Reference Laboratory, Lanzhou Veterinary Research Institute, Chinese Academy of Agricultural Sciences. For the virus infection experiment, PK-15 cells were washed with phosphate-buffered saline (PBS) and then incubated with strain O/BY/CHA/2010 at a multiplicity of infection (MOI) of 0.5. The cells were maintained at 37°C for 1 h, and the inocula were then removed after the adsorption course. The infected cells were cultured in viral maintenance media containing 1% FBS for the indicated times. The infected cells were collected and subjected to appropriate experiments.

### Plasmid constructs and transfection.

The full-length porcine RPSA, VIM, ESD, and TPM4 cDNAs were amplified from PK-15 cells and constructed into pcDNATM3.1/myc-His(–)A vector (Invitrogen, Carlsbad, CA) to generate plasmids expressing Myc-tagged RPSA (Myc-RPSA), VIM (Myc-VIM), ESD (Myc-ESD), and TPM4 (Myc-TPM4), respectively. The Flag-VP1 expressing plasmid was constructed previously by our laboratory (3). A series of Flag-tagged truncated VP1 constructs (Flag-VP1-Δ1-35, Flag-VP1-Δ30-65, Flag-VP1-Δ60-95, Flag-VP1-Δ90-125, Flag-VP1-Δ120-155, Flag-VP1-Δ150-185, Flag-VP1-Δ180-214, Flag-VP1-1-115, Flag-VP1-115-214, and Flag-VP1-37-188) were generated by site-directed mutagenesis PCR, as described previously (41). All of the constructed plasmids were sequenced and analyzed to ensure the accurate insertion of the target genes into the vector plasmids. Lipofectamine 2000 (Invitrogen) was used as the transfection reagent. All of the transfection experiments were performed according to the manufacturer’s instructions.

### Quantitative PCR.

Total RNA from the collected samples was extracted using TRIzol reagent (Invitrogen) according to the protocol provided by the manufacturer. The first-strand cDNA was synthesized using the Moloney murine leukemia virus reverse transcriptase and random hexamer primers (TaKaRa, Dalian, China), using the extracted RNA as the templates. Expression analysis of all of the target genes was performed by quantitative PCR (qPCR). The qPCR was performed using SYBR Premix ExTaq reagents (TaKaRa) on a QuantStudio 5 real-time PCR system (Applied Biosystems, Foster City, CA) according to the manufacturer’s instructions. The glyceraldehyde 3-phosphate dehydrogenase (GAPDH) gene was used as an internal control in the gene expression studies. The relative amounts of each transcript were calculated by using the comparative cycle threshold (2^−ΔΔ^*^CT^*) method (42). All of the experiments were repeated three independent times, with similar results. The data represent results from one representative triplicate experiment.

### Immunoblotting analysis.

For Western blotting, the cells were lysed in a lysis buffer described previously (43). The cell lysates were briefly sonicated and boiled, and the cell debris was removed by centrifugation at 20,000 × *g* at 4°C for 10 min. The supernatant was electrophoresed according to a standard protocol. The target proteins were transferred onto nitrocellulose transfer membranes (Pall Crop, East Hills, NY). The membranes were subsequently incubated for 4 h at room temperature in 5% skim milk and incubated with appropriate primary and secondary antibodies. Antigen-antibody complexes were visualized by enhanced chemiluminescence (Amersham Pharmacia Biotech, Piscataway, NJ).

### RNA interference.

siRNA was used to knockdown cellular RPSA expression. The transfection of siRNA was performed using Lipofectamine 2000 (Invitrogen) as described previously (3). PK-15 cells were cultured in 6-well plates, and the monolayer cells were transfected with 120 nM NC siRNA or siRNAs that target RPSA (RPSA siRNA) using Lipofectamine 2000. The cells were subjected to other experiments at 36 h posttransfection. The porcine RPSA siRNA sequences included siRNA-1 (forward, CCAUCGUUGCCAUUGAAAATT; reverse, UUUUCAAUGGCAACGAUGGTT) and siRNA-2 (forward, CCAUCCCGUGCAACAACAATT; reverse, UUGUUGUUGCACGGGAUGGTT).

### Coimmunoprecipitation assay.

HEK-293T cells were cultured in 10-cm dishes, and the monolayer cells were cotransfected with the indicated plasmids. The transfected cells were washed with PBS and lysed with 500 μl of lysis buffer. The lysates were subjected to the immunoprecipitation experiment as described previously using appropriate antibodies (29). As for the immunoprecipitation of RPSA with VP1 in the context of viral infection, PK-15 cells were cultured in 10-cm dishes, and the monolayer cells were mock infected or infected with FMDV at an MOI of 0.5 for 12 h. The cell lysates were immunoprecipitated with anti-RPSA antibody and subjected to Western blotting. For membrane protein detection, the cell membrane proteins were extracted using a Mem-PER Plus membrane protein extraction kit (Thermo Scientific) according to the manufacturer’s protocol. The membrane fractions were then immunoprecipitated with anti-RPSA or anti-Myc antibody and subjected to Western blotting.

### Indirect immunofluorescence assay.

HEK-293T or PK-15 cells were seeded on Nunc glass-bottom dishes for 12 h, followed by transfection or infection as indicated. The transfected or infected cells were fixed and permeabilized by a acetone-methanol mixture (1:1) for 10 min at –20°C. Nonspecific binding was blocked with 5% normal goat serum in PBS for 1 h at room temperature before incubation at 4°C overnight with different primary antibodies. The fluorochrome-conjugated secondary antibodies were then used for staining the specimens to visualize VP1 or RPSA proteins. Nuclei were visualized using DAPI (4′,6′-diamidino-2-phenylindole). Stains were evaluated with a confocal Nikon eclipse 80i fluorescence microscope with appropriate settings. The microscopy images were processed using NIS Elements F 2.30 software.

### Statistical analysis.

The significance of the results between the experiments was analyzed using Prism 5.0 software (GraphPad, San Diego, CA). The data are presented as means ± the standard deviations (SD). The criterion *P* value for statistical significance was <0.05 (*, *P* < 0.05 [significant]; **, *P* < 0.01 [highly significant]).
